# Supplementary material for: A mediation analysis evaluating change in self-stigma on diabetes outcomes among people with depression in urban India: A secondary analysis from the INDEPENDENT trial of the collaborative care model
Source: PLOS Glob Public Health. 2024 Sep 4;4(9):e0003624. doi: 10.1371/journal.pgph.0003624 (PMC11373850; doi:10.1371/journal.pgph.0003624)
Supplement: S1 File — (DOCX) [file pgph.0003624.s002.docx]

### S1 File: 4-item Self-Stigma Scale for Chronic Illness

| PART – B: STIGMA SCALE FOR CHRONIC ILLNESS | | | | | |
| --- | --- | --- | --- | --- | --- |
| The following items ask about any stigma (shame, embarrassment) you have experienced lately as a result of your condition. | | | | | |
| Please indicate which condition (diabetes/sugar, depression/stress) is most stigmatizing for you | Depression  Diabetes  *Please consider this condition when answering the following questions.* | | | | |
|  | **Never (1)** | **Rarely (2)** | **Sometimes (3)** | **Often (4)** | **Always (5)** |
| Because of my illness, some people avoided me |  |  |  |  |  |
| Some people acted as though it was my fault I have this illness |  |  |  |  |  |
| Because of my illness, I felt left out of things |  |  |  |  |  |
| I felt embarrassed about my illness |  |  |  |  |  |
