# Supplementary material for: A mediation analysis evaluating change in self-stigma on diabetes outcomes among people with depression in urban India: A secondary analysis from the INDEPENDENT trial of the collaborative care model
Source: PLOS Glob Public Health. 2024 Sep 4;4(9):e0003624. doi: 10.1371/journal.pgph.0003624 (PMC11373850; doi:10.1371/journal.pgph.0003624)
Supplement: S2 File — (DOCX) [file pgph.0003624.s003.docx]

### S2 File – Annotated statistical software code

## This is annotated companion R code for:

## Halliday S, Rao D, Augusto O, et al. A mediation analysis

## evaluating change in self-stigma on diabetes outcomes among

## people with depression in urban India: A secondary analysis

## from the INDEPENDENT trial of the Collaborative Care Model.

## Description:

## This R code provides a complete walk through of this mediation analysis

## according to the Baron & Kenny (1986) method with bootstrapping and

## multiple imputation.

## Data availability:

## To obtain the datasets to reproduce this analysis, please email

## the Emory Global Diabetes Research Center: egdrc@emory.edu You will be asked to enter into a data

## sharing agreement with co-investigators for the INDEPENDENT study.

## Trial registration:

## https://clinicaltrials.gov/ct2/show/NCT02022111

## This R code is divided into the following sections:

## 1) Data preparation and set-up

## 2) Construct validity

## 3) Missingness exploration and multiple imputations for mediation analysis

## 4) Mediation analysis (unadjusted and adjusted models)

## 5) Longitudinal analysis of self-stigma scores (SSCI) by treatment group

## (data preparation and set up; multiple imputations for longitudinal analysis;

## longitudinal analysis of SSCI by treatment group)

## Session Info

## R version 4.2.2 (2022-10-31 ucrt)

## Platform: x86_64-w64-mingw32/x64 (64-bit)

## Running under: Windows 10 x64 (build 22621)

## Packages used to perform analysis (if you do not have these, they can

## be installed using the following code:

## install.packages("[insert package name"]))

##### data preparation set up for analyses #####

## packages used

library(tidyverse)

library(table1)

library(Amelia)

library(lattice)

library(mice)

library(VIM)

library(mediation)

library(nlme)

library(mitools)

library(mitml)

library(broom.mixed)

library(data.table)

library(broom)

library(psych)

## set data directory; change root to correspond with location where data is saved

data.dir <- "C:/Users/smhal/Documents/University of Washington/PhD 2019-2023/Dissertation/Aim 3/"

setwd(data.dir)

## set seed for reproducible results; choose a random number

set.seed(71086)

## load data, ##0mo and 12mo used for analysis

INDEP_0mo <- read.csv('Complete_INDEP_NEW_0mo.csv', header=TRUE) ##for mediation + longitudinal stigma analysis

INDEP_12mo <- read.csv('Complete_INDEP_NEW_12mo.csv', header=TRUE) ##for mediation + longitudinal stigma analysis

INDEP_24mo <- read.csv('Complete_INDEP_NEW_24mo.csv', header=TRUE) ##longitudinal stigma analysis only; supplemental

INDEP_36mo <- read.csv('Complete_INDEP_NEW_36mo.csv', header=TRUE, fileEncoding="latin1") ##longitudinal stigma analysis only; supplemental

## set BL model for predictors/outcomes of interest only

mod_bl <- PID ~ PType + SID + Age + Sex + edustat + hh_inc + PHQ9_tot + A1C + Stigma_cond + Stigma1 + Stigma2 + Stigma3 + Stigma4

## set endline model

mod_end <- PID ~ PHQ9_tot + A1C + Stigma1 + Stigma2 + Stigma3 + Stigma4

## create new dataframes with only predictors/outcomes of interest

INDEP_0mo_fin <- model.frame(mod_bl, data=INDEP_0mo, na.action = na.pass)

INDEP_12mo_fin <- model.frame(mod_end, data=INDEP_12mo, na.action = na.pass)

## create new education status (below primary, primary complete, secondary complete, post-secondary)

INDEP_0mo_fin <- INDEP_0mo_fin %>% mutate(educ = case_when(

INDEP_0mo_fin$edustat==7~0,

INDEP_0mo_fin$edustat==6~1,

INDEP_0mo_fin$edustat==5~1,

INDEP_0mo_fin$edustat==4~2,

INDEP_0mo_fin$edustat==3~3,

INDEP_0mo_fin$edustat==2~4,

INDEP_0mo_fin$edustat==1~4,

INDEP_0mo_fin$edustat==0~4

))

## create NAs for educ == 0 (unsure)

INDEP_0mo_fin$educ[INDEP_0mo_fin$educ=="0"] <- NA

## join follow-up observations into single dataframe

INDEP <- left_join(INDEP_0mo_fin, INDEP_12mo_fin, by="PID")

## reclassify 0 values for stigma as NAs

INDEP[, 11:14][INDEP[, 11:14] == 0] <- NA

INDEP[, 18:21][INDEP[, 18:21] == 0] <- NA

## rows 263; 405-401 to be deleted; 363 had A1c value of 0 and was removed from analysis, 405-411 extra rows in original dataset

INDEP <- INDEP[-c(363,405,406,407,408,409,410,411), ]

## remove edustat variable

INDEP <- INDEP[ -c(6) ]

## create new total stigma scores

INDEP <- INDEP %>% mutate(tot_stigma.x = Stigma1.x+Stigma2.x+Stigma3.x+Stigma4.x)

INDEP <- INDEP %>% mutate(tot_stigma.y = Stigma1.y+Stigma2.y+Stigma3.y+Stigma4.y)

## create change in stigma scores as primary predictor

INDEP <- INDEP %>% mutate(stigma_change = tot_stigma.y-tot_stigma.x)

##### construct validity #####

## stigma scale variables

## use baseline measurement for cronbach alpha

stigma.vars <- c("Stigma1.x", "Stigma2.x", "Stigma3.x", "Stigma4.x")

## run Cronbahch's alpha on stigma scale variables total

stigma.alpha <- alpha(INDEP[stigma.vars])

print(stigma.alpha, digits=3)

##### descriptive characteristics for mediation analysis #####

## categorical age

INDEP <- INDEP %>% mutate(age_category = case_when(

INDEP$Age<30 ~ 'age <30 years',

INDEP$Age>=30 & INDEP$Age<40 ~ 'age 30-39 years',

INDEP$Age>=40 & INDEP$Age<50 ~ 'age 40-49 years',

INDEP$Age>=50 & INDEP$Age<60 ~ 'age 50-59 years',

INDEP$Age>=60 ~"age>= 60 years"))

## factor variables/descriptive labels for table 1 based on imputations

INDEP$PType <-

factor(INDEP$PType, levels=c(0,1),

labels=c("Control",

"Intervention"))

label(INDEP$PType) <- "Treatment Assignment"

## optional if interested to look at table stratified by site

INDEP$SID <-

factor(INDEP$SID, levels=c(1,2,3,4),

labels=c("MDRF",

"AIIMS",

"Vizag",

"Diacon"))

INDEP$Sex <-

factor(INDEP$Sex, levels=c(0,1),

labels=c("Male",

"Female"))

INDEP$educ <-

factor(INDEP$educ, levels=c(1,2,3,4),

labels=c("Less than primary school",

"Primary school",

"Secondary school",

"Post-secondary school"))

label(INDEP$educ) <- "Level of educational attainment"

INDEP$hh_inc <-

factor(INDEP$hh_inc, levels=c(1,2,3,4,5,6,7),

labels=c("<3,000INRs",

"3,000-10,000INRs",

"10,001-20,000INRs",

"20,001-30,000INRs",

"30,001-40,000INRs",

"40,001-50,000INRs",

">50,000INRs"))

label(INDEP$hh_inc) <- "Average monthly household income (INRs)"

INDEP$Stigma_cond <-

factor(INDEP$Stigma_cond, levels=c(1,2),

labels=c("Depression",

"Diabetes"))

label(INDEP$Stigma_cond) <- "Stigmatized condition"

label(INDEP$stigma_change) <- "Change in Self-Stigma score (SSCI; range: -16 - +16)"

label(INDEP$PHQ9_tot.x) <- "Depression at baseline (PHQ9; range: 0-27)"

label(INDEP$PHQ9_tot.y) <- "Depression at endline (PHQ9; range: 0-27)"

label(INDEP$A1C.x) <- "Diabetes at baseline (A1c)"

label(INDEP$A1C.y) <- "Diabetes at endline (A1c)"

label(INDEP$tot_stigma.x) <- "Self-Stigma score at baseline (SSCI; range: 4-20)"

label(INDEP$age_category) <- "Categorical Age"

## table 1 pre-imputation

## configure table 1 for IQR

my.render.median.IQR <- function(x, ...) {

with(stats.apply.rounding(stats.default(x, ), digits = 3),

c("",

"Median [IQR]" =

sprintf(paste("%s [",Q1,"- %s]"), MEDIAN,Q3)))

}

table1_pre_imp <- table1(~ Sex + age_category + educ + hh_inc + as.factor(Stigma_cond) + tot_stigma.x + stigma_change + PHQ9_tot.x + PHQ9_tot.y + A1C.x + A1C.y | PType, data=INDEP, render.continuous=my.render.median.IQR)

table1_pre_imp

## table 1 pre-imputation, stratified by site, not used

table1_pre_imp_site <- table1(~ Sex + Age + educ + hh_inc + as.factor(Stigma_cond) + tot_stigma.x + stigma_change + PHQ9_tot.x + PHQ9_tot.y + A1C.x + A1C.y | factor(SID) + PType, data=INDEP, render.continuous=my.render.median.IQR)

table1_pre_imp_site

##### missingness exploration and multiple imputations for mediation analysis #####

## exploratory analysis for missingness

INDEP_miss = aggr(INDEP, col=mdc(1:2), numbers=TRUE, sortVars=TRUE, labels=names(INDEP), cex.axis=.5, gap=2, ylab=c("Proportion of missingness","Missingness Pattern"))

## drop individual stigma scores and total stigma score to avoid collinearity with stigma outcomes

INDEP <- INDEP[ -c(10:13) ] ##baseline stigma scores

INDEP <- INDEP[ -c(13:18) ] ##endline stigma scores and total stigma scores

## create bounds for PHQ9 imputations (restrict to values of 0-27)

bds <- matrix(c(11,0,27), nrow=1, ncol=3)

bds ##check that it worked

## use AMELIA for 30 multiple imputations

set.seed(71086)

m = 30

INDEP_imp <- amelia(INDEP, m=m, p2s = 1,

idvars=c("PID","PType","SID","age_category"),

noms=c("Stigma_cond"),

ords=c("educ", "Sex", "hh_inc"),

max.resample = 1000,

bounds = bds)

INDEP_imp

plot(INDEP_imp) ## assess convergence ## education variable has multiple peaks indicating overdispersion

write.amelia(obj=INDEP_imp, file.stem="INDEP_imp")

## set up imputed datasets for mediation analysis, store as list-like object

setwd(data.dir)

ll.imp_sets <-

lapply(1:m, function(i){

INDEP_imp.i <- read.csv(paste0('indep_imp', i, '.csv'), header=TRUE) ## load m=30 imputed datasets

INDEP_imp.i$PHQ9_tot.y <-round(INDEP_imp.i$PHQ9_tot.y) ## round imputed PHQ9 values to nearest whole numbers (no missingness at baseline)

INDEP_imp.i$stigma_change <- round(INDEP_imp.i$stigma_change) ## round imputed stigma change values to nearest whole number

INDEP_imp.i$A1C.y <- round(INDEP_imp.i$A1C.y, digits = 1) ## round imputed A1c values to 1 decimal place (no missingness at baseline)

return(INDEP_imp.i)

})

## check an imputed dataset

INDEP_imp1 <- ll.imp_sets[[1]]

## table 1 for selected imputed dataset to check for missingness

table1_post_imp <- table1(~ Sex + age_category + educ + hh_inc + as.factor(Stigma_cond) + stigma_change + PHQ9_tot.x + PHQ9_tot.y + A1C.x + A1C.y | PType, data=INDEP_imp1, render.continuous=my.render.median.IQR)

table1_post_imp

## check for no missingness

##### evaluate data distributions for mediation analysis #####

## density plot depression (use imputed dataset 1 for reference)

## call dev.off if invalid graphics state error persists

dens_dep_12mo <- ggplot(INDEP_imp1,

aes(x=PHQ9_tot.y)) +

geom_density(fill="#69b3a2", color="#e9ecef", alpha=0.8) +

theme_light() +

labs(

title = "Distribution of depression at endline",

x = "Depression at endline (PHQ9 scores, range: 0-27)",

y = "Distribution of depression at endline")

dens_dep_12mo

dens_dep_0mo <- ggplot(INDEP_imp1,

aes(x=PHQ9_tot.x)) +

geom_density(fill="#69b3a2", color="#e9ecef", alpha=0.8) +

theme_light() +

labs(

title = "Distribution of depression at baseline",

x = "Depression at endline (PHQ9 scores, range: 0-27)",

y = "Distribution of depression at baseline")

dens_dep_0mo

## density plot diabetes endline

dens_diab_12mo <- ggplot(INDEP_imp1,

aes(x=A1C.y)) +

geom_density(fill="#69b3a2", color="#e9ecef", alpha=0.8) +

theme_light() +

labs(

title = "Distribution of diabetes at endline",

x = "Diabetes at endline (glycated hemoglobin A1c scores,0-100%",

y = "Distribution of diabetes at endline")

dens_diab_12mo

dens_diab_0mo <- ggplot(INDEP_imp1,

aes(x=A1C.x)) +

geom_density(fill="#69b3a2", color="#e9ecef", alpha=0.8) +

theme_light() +

labs(

title = "Distribution of diabetes at baseline",

x = "Diabetes at baseline (glycated hemoglobin A1c scores,0-100%",

y = "Distribution of diabetes at baseline")

dens_diab_0mo

## convert multiple imputed datsets to mids object

INDEP_mids <- miceadds::datalist2mids(INDEP_imp$imputations)

##### mediation analysis #####

### unadjusted analysis ###

## change in stigma = predictor, diabetes = outcome, no mediator

mod.0_imp <- A1C.y ~ stigma_change + A1C.x

## change in stigma = predictor, depression = mediator, no outcome

mod.1_imp <- PHQ9_tot.y ~ stigma_change + PHQ9_tot.x

## change in stigma = predictor, depression = mediator, diabetes = outcome, full model

mod.2_imp <- A1C.y ~ stigma_change + A1C.x + PHQ9_tot.x + PHQ9_tot.y

## bootstrapped mediation results, save as list-like object

ll.results <-

lapply(1:m, function(i) {

mod.0_outp_imp <- lm(mod.0_imp, data = ll.imp_sets[[i]]) ## change in stigma = predictor, diabetes = outcome, no mediator

mod.1_outp_imp <- lm(mod.1_imp, data = ll.imp_sets[[i]]) ## change in stigma = predictor, depression = mediator, no outcome

mod.2_outp_imp <- lm(mod.2_imp, data = ll.imp_sets[[i]]) ## change in stigma = predictor, depression = mediator, diabetes = outcome, full model

results_med <- mediation::mediate(mod.1_outp_imp,

mod.2_outp_imp, treat = 'stigma_change', mediator = 'PHQ9_tot.y', boot = TRUE, sims = 1000) ## bootstrap CI with 1000 simulations

list("mod.0_outp_imp" = mod.0_outp_imp,

"mod.1_outp_imp" = mod.1_outp_imp,

"mod.2_outp_imp" = mod.2_outp_imp,

"results_med" = results_med)

})

save(list = c("ll.imp_sets", "ll.results"), file = "imputed_datasets_results.image")

load("imputed_datasets_results.image")

## pooled mediation results

## bootstrapped p-values function, adapted from boot.pval::boot.pval[]

modified.boot.pval <- function(sims, theta_null = 0) {

pval_precision = 1/length(sims)

alpha_seq <- seq(1e-16, 1 - 1e-16, pval_precision)

ci <- lapply(alpha_seq, function(xalpha) {rbind(quantile(sims, c(xalpha/2, 1 - xalpha/2)))})

bounds <- do.call(rbind, ci)

alpha <- alpha_seq[which.min(theta_null >= bounds[,1] & theta_null <= bounds[,2])]

return(alpha)

}

## p values function from mediation package

p.val <- function (x, xhat){

if (xhat == 0)

out <- 1

else {

out <- 2 * min(sum(x > 0), sum(x < 0))/length(x)

}

return(min(out, 1))

}

## ACME

## Pooled ACME point estimate

ll.med.results <- lapply(1:m, function(i){ll.results[[i]]$results_med})

pool.ACME <-

(ll.m <-

lapply(ll.med.results,

function(l){

l$d0

}) %>% unlist()) %>% mean()

pool.ACME

## Pooled ACME CI

pool.ACME.CI <-

(ll.ACME <-

lapply(lapply(1:m, function(i){ll.results[[i]]$results_med}),

function(l) {

l$d0.sims

}) %>% unlist()) %>% quantile(., c(2.5, 97.5)/100)

pool.ACME.CI

## Pooled ACME p value

plot(density(ll.ACME[1:1000]))

plot(density(ll.ACME[1:1000 + 1000]))

plot(density(ll.ACME[1:1000 + 2*1000]))

plot(density(ll.ACME[1:1000 + 20*1000]))

plot(density(ll.ACME[1:1000 + 29*1000]))

pool.ACME.pval <- p.val(ll.ACME, xhat = pool.ACME)

pool.ACME.pval

pool.ACME.pval.al <- modified.boot.pval(ll.ACME)

pool.ACME.pval.al

## ADE

## Pooled ADE point estimate

pool.ADE <-

lapply(lapply(1:m, function(i){ll.results[[i]]$results_med}),

function(l) {l$z0}) %>% unlist() %>% mean()

pool.ADE

## Pooled ADE CI

pool.ADE.CI <-

(ll.ADE <-

lapply(lapply(1:m, function(i){ll.results[[i]]$results_med}),

function(l) {

l$z0.sims

}) %>% unlist()

) %>% quantile(., c(2.5, 97.5)/100)

pool.ADE.CI

## Pooled ADE p value

pool.ADE.pval <- p.val(ll.ADE, xhat = pool.ADE)

pool.ADE.pval

pool.ADE.pval.al <- modified.boot.pval(ll.ADE)

pool.ADE.pval.al

## Pooled total effect

pool.Tot <-

lapply(lapply(1:m, function(i){ll.results[[i]]$results_med}),

function(l) {l$tau.coef}) %>% unlist() %>% mean()

pool.Tot

## Pooled total effect CI

pool.Tot.CI <-

(ll.Tot <-

lapply(lapply(1:m, function(i){ll.results[[i]]$results_med}),

function(l) {

l$tau.sims

}) %>% unlist()) %>% quantile(., c(2.5, 97.5)/100)

pool.Tot.CI

## Pooled total effect p value

pool.Tot.pval <- p.val(ll.Tot, xhat = pool.Tot)

pool.Tot.pval

pool.Tot.pval.al <- modified.boot.pval(ll.Tot)

pool.Tot.pval.al

## Prop. Mediated

## Prop. Mediated point estimate

pool.Prop.Med <-

lapply(lapply(1:m, function(i){ll.results[[i]]$results_med}),

function(l) {l$n0}) %>% unlist() %>% mean()

pool.Prop.Med

## Prop. Mediated CI

pool.Prop.Med.CI <-

(ll.Med <-

lapply(lapply(1:m, function(i){ll.results[[i]]$results_med}),

function(l) {

l$n0.sims

}) %>% unlist()) %>% quantile(., c(2.5, 97.5)/100)

pool.Prop.Med.CI

## Prop. Mediate p value

pool.Prop.Med.pval <- p.val(ll.Med, xhat = pool.Prop.Med)

pool.Prop.Med.pval

pool.Prop.Med.pval.al <- modified.boot.pval(ll.Med)

pool.Prop.Med.pval.al

## Store results in tibble

TAB <- ## With p.val() p-values

tribble(

~indicator, ~Estimate, ~LB, ~UB, ~pval,

"ACME", pool.ACME, pool.ACME.CI[1], pool.ACME.CI[2], pool.ACME.pval,

"ADE", pool.ADE, pool.ADE.CI[1], pool.ADE.CI[2], pool.ADE.pval,

"Total Effect", pool.Tot, pool.Tot.CI[1], pool.Tot.CI[2], pool.Tot.pval,

"Prop. Mediated", pool.Prop.Med, pool.Prop.Med.CI[1], pool.Prop.Med.CI[2], pool.Prop.Med.pval

)

TAB

TAB1 <- ## With p.val() and boot.pval() p-values

tribble(

~indicator, ~Estimate, ~LB, ~UB, ~pval, ~pval.al,

"ACME", pool.ACME, pool.ACME.CI[1], pool.ACME.CI[2], pool.ACME.pval, pool.ACME.pval.al,

"ADE", pool.ADE, pool.ADE.CI[1], pool.ADE.CI[2], pool.ADE.pval, pool.ADE.pval.al,

"Total Effect", pool.Tot, pool.Tot.CI[1], pool.Tot.CI[2], pool.Tot.pval, pool.Tot.pval.al,

"Prop. Mediated", pool.Prop.Med, pool.Prop.Med.CI[1], pool.Prop.Med.CI[2], pool.Prop.Med.pval, pool.Prop.Med.pval

)

TAB1

### adjusted analysis ###

## model formulas

## change in stigma = predictor, diabetes = outcome, no mediator

mod.0_imp_adj <- A1C.y ~ stigma_change + A1C.x + Age + Sex + hh_inc + educ + PType

## change in stigma = predictor, depression = mediator, no outcome

mod.1_imp_adj <- PHQ9_tot.y ~ stigma_change + PHQ9_tot.x + Age + Sex + hh_inc + educ + PType

## change in stigma = predictor, depression = mediator, diabetes = outcome, full model

mod.2_imp_adj <- A1C.y ~ stigma_change + A1C.x + PHQ9_tot.x + PHQ9_tot.y + Age + Sex + hh_inc + educ + PType

## bootstrapped mediation (adjusted) results, save as list-like object

ll.results_adj <-

lapply(1:m, function(i) {

mod.0_outp_imp_adj <- lm(mod.0_imp_adj, data = ll.imp_sets[[i]]) ## change in stigma = predictor, diabetes = outcome, no mediator

mod.1_outp_imp_adj <- lm(mod.1_imp_adj, data = ll.imp_sets[[i]]) ## change in stigma = predictor, depression = mediator, no outcome

mod.2_outp_imp_adj <- lm(mod.2_imp_adj, data = ll.imp_sets[[i]]) ## change in stigma = predictor, depression = mediator, diabetes = outcome, full model

results_med_adj <- mediation::mediate(mod.1_outp_imp_adj,

mod.2_outp_imp_adj, treat = 'stigma_change', mediator = 'PHQ9_tot.y', boot = TRUE, sims = 1000) ## bootstrap CI with 1000 simulations

list("mod.0_outp_imp_adj" = mod.0_outp_imp_adj,

"mod.1_outp_imp_adj" = mod.1_outp_imp_adj,

"mod.2_outp_imp_adj" = mod.2_outp_imp_adj,

"results_med_adj" = results_med_adj)

})

save(list = c("ll.imp_sets", "ll.results_adj"), file = "imputed_datasets_results_adj.image")

load("imputed_datasets_results_adj.image")

## pooled mediation results, adjusted analysis

## bootstrapped p-values function, adapted from boot.pval::boot.pval[]

modified.boot.pval <- function(sims, theta_null = 0) {

pval_precision = 1/length(sims)

alpha_seq <- seq(1e-16, 1 - 1e-16, pval_precision)

ci <- lapply(alpha_seq, function(xalpha) {rbind(quantile(sims, c(xalpha/2, 1 - xalpha/2)))})

bounds <- do.call(rbind, ci)

alpha <- alpha_seq[which.min(theta_null >= bounds[,1] & theta_null <= bounds[,2])]

return(alpha)

}

## p values function from mediation package

p.val <- function (x, xhat){

if (xhat == 0)

out <- 1

else {

out <- 2 * min(sum(x > 0), sum(x < 0))/length(x)

}

return(min(out, 1))

}

## ACME

## Pooled ACME point estimate

ll.med.results_adj <- lapply(1:m, function(i){ll.results_adj[[i]]$results_med_adj})

pool.ACME_adj <-

(ll.m_adj <-

lapply(ll.med.results_adj,

function(l){

l$d0

}) %>% unlist()) %>% mean()

pool.ACME_adj

## Pooled ACME CI

pool.ACME.CI_adj <-

(ll.ACME_adj <-

lapply(lapply(1:m, function(i){ll.results_adj[[i]]$results_med_adj}),

function(l) {

l$d0.sims

}) %>% unlist()) %>% quantile(., c(2.5, 97.5)/100)

pool.ACME.CI_adj

## Pooled ACME p value

plot(density(ll.ACME_adj[1:1000]))

plot(density(ll.ACME_adj[1:1000 + 1000]))

plot(density(ll.ACME_adj[1:1000 + 2*1000]))

plot(density(ll.ACME_adj[1:1000 + 20*1000]))

plot(density(ll.ACME_adj[1:1000 + 29*1000]))

pool.ACME.pval_adj <- p.val(ll.ACME_adj, xhat = pool.ACME_adj)

pool.ACME.pval_adj

pool.ACME.pval.al_adj <- modified.boot.pval(ll.ACME_adj)

pool.ACME.pval.al_adj

## ADE

## Pooled ADE point estimate

pool.ADE_adj <-

lapply(lapply(1:m, function(i){ll.results_adj[[i]]$results_med_adj}),

function(l) {l$z0}) %>% unlist() %>% mean()

pool.ADE_adj

## Pooled ADE CI

pool.ADE.CI_adj <-

(ll.ADE_adj <-

lapply(lapply(1:m, function(i){ll.results_adj[[i]]$results_med_adj}),

function(l) {

l$z0.sims

}) %>% unlist()

) %>% quantile(., c(2.5, 97.5)/100)

pool.ADE.CI_adj

## Pooled ADE p value

pool.ADE.pval_adj <- p.val(ll.ADE, xhat = pool.ADE_adj)

pool.ADE.pval_adj

pool.ADE.pval.al_adj <- modified.boot.pval(ll.ADE_adj)

pool.ADE.pval.al_adj

## Pooled total effect

pool.Tot_adj <-

lapply(lapply(1:m, function(i){ll.results_adj[[i]]$results_med_adj}),

function(l) {l$tau.coef}) %>% unlist() %>% mean()

pool.Tot_adj

## Pooled total effect CI

pool.Tot.CI_adj <-

(ll.Tot_adj <-

lapply(lapply(1:m, function(i){ll.results_adj[[i]]$results_med_adj}),

function(l) {

l$tau.sims

}) %>% unlist()) %>% quantile(., c(2.5, 97.5)/100)

pool.Tot.CI_adj

## Pooled total effect p value

pool.Tot.pval_adj <- p.val(ll.Tot, xhat = pool.Tot_adj)

pool.Tot.pval_adj

pool.Tot.pval.al_adj <- modified.boot.pval(ll.Tot_adj)

pool.Tot.pval.al_adj

## Prop. Mediated

## Prop. Mediated point estimate

pool.Prop.Med_adj <-

lapply(lapply(1:m, function(i){ll.results_adj[[i]]$results_med_adj}),

function(l) {l$n0}) %>% unlist() %>% mean()

pool.Prop.Med_adj

## Prop. Mediated CI

pool.Prop.Med.CI_adj <-

(ll.Med_adj <-

lapply(lapply(1:m, function(i){ll.results_adj[[i]]$results_med_adj}),

function(l) {

l$n0.sims

}) %>% unlist()) %>% quantile(., c(2.5, 97.5)/100)

pool.Prop.Med.CI_adj

## Prop. Mediate p value

pool.Prop.Med.pval_adj <- p.val(ll.Med, xhat = pool.Prop.Med_adj)

pool.Prop.Med.pval_adj

pool.Prop.Med.pval.al_adj <- modified.boot.pval(ll.Med_adj)

pool.Prop.Med.pval.al_adj

## Store results in tibble

TAB_adj <- ## With p.val() p-values

tribble(

~indicator, ~Estimate, ~LB, ~UB, ~pval,

"ACME", pool.ACME_adj, pool.ACME.CI_adj[1], pool.ACME.CI_adj[2], pool.ACME.pval_adj,

"ADE", pool.ADE_adj, pool.ADE.CI_adj[1], pool.ADE.CI_adj[2], pool.ADE.pval_adj,

"Total Effect", pool.Tot_adj, pool.Tot.CI_adj[1], pool.Tot.CI_adj[2], pool.Tot.pval_adj,

"Prop. Mediated", pool.Prop.Med_adj, pool.Prop.Med.CI_adj[1], pool.Prop.Med.CI_adj[2], pool.Prop.Med.pval_adj

)

TAB_adj

TAB1_adj <- ## With p.val() and boot.pval() p-values

tribble(

~indicator, ~Estimate, ~LB, ~UB, ~pval, ~pval.al,

"ACME", pool.ACME_adj, pool.ACME.CI_adj[1], pool.ACME.CI_adj[2], pool.ACME.pval_adj, pool.ACME.pval.al_adj,

"ADE", pool.ADE_adj, pool.ADE.CI_adj[1], pool.ADE.CI_adj[2], pool.ADE.pval_adj, pool.ADE.pval.al_adj,

"Total Effect", pool.Tot_adj, pool.Tot.CI_adj[1], pool.Tot.CI_adj[2], pool.Tot.pval_adj, pool.Tot.pval.al_adj,

"Prop. Mediated", pool.Prop.Med_adj, pool.Prop.Med.CI_adj[1], pool.Prop.Med.CI_adj[2], pool.Prop.Med.pval_adj, pool.Prop.Med.pval_adj

)

TAB1_adj

##### longitudinal analysis of SSCI by treatment group #####

### data preparation and set up ###

## set longitudinal model

mod_long <- PID ~ Stigma1 + Stigma2 + Stigma3 + Stigma4

## create data frames with only model; 24mo and 36mo are supplemental and not used

INDEP_24mo_fin <- model.frame(mod_long, data=INDEP_24mo, na.action = na.pass)

INDEP_36mo_fin <- model.frame(mod_long, data=INDEP_36mo, na.action = na.pass)

## reclassify 0 values for stigma as NAs

INDEP_24mo_fin[, 2:5][INDEP_24mo_fin[, 2:5] == 0] <- NA

INDEP_36mo_fin[, 2:5][INDEP_36mo_fin[, 2:5] == 0] <- NA

## mutate new total stigma scores in longitudinal data frames

INDEP_24mo_fin <- INDEP_24mo_fin %>% mutate(tot_stigma.24mo = Stigma1+Stigma2+Stigma3+Stigma4)

INDEP_36mo_fin <- INDEP_36mo_fin %>% mutate(tot_stigma.36mo = Stigma1+Stigma2+Stigma3+Stigma4)

## join dataframes

INDEP_long <- left_join(INDEP_0mo_fin, INDEP_12mo_fin, by="PID")

## rows 363, 405-4011 to be deleted; 363 had A1c value of 0 and was removed from analysis, 405-411 extra rows in original dataset

INDEP_long <- INDEP_long[-c(363,405,406,407,408,409,410,411), ]

## join dataframes

INDEP_long <- left_join(INDEP_long, INDEP_24mo_fin, by="PID")

INDEP_long <- left_join(INDEP_long, INDEP_36mo_fin, by="PID")

## mutate new total stigma scores from baseline/12mo

INDEP_long <- INDEP_long %>% mutate(tot_stigma.0mo = Stigma1.x+Stigma2.x+Stigma3.x+Stigma4.x)

INDEP_long <- INDEP_long %>% mutate(tot_stigma.12mo = Stigma1.y+Stigma2.y+Stigma3.y+Stigma4.y)

## drop unused variables for longitudinal analysis

INDEP_long <- INDEP_long[ -c(11:14) ]

INDEP_long <- INDEP_long[ -c(14:21) ]

INDEP_long <- INDEP_long[ -c(15:18) ]

## convert to long format for repeated measures indexed by time

INDEP_long <- INDEP_long %>%

gather(key = "time", value = "SSCI", tot_stigma.0mo, tot_stigma.12mo, tot_stigma.24mo, tot_stigma.36mo)

## relabel time values

INDEP_long$time <-

factor(INDEP_long$time, levels=c("tot_stigma.0mo","tot_stigma.12mo","tot_stigma.24mo","tot_stigma.36mo"),

labels=c("0",

"12",

"24",

"36"))

### multiple imputations for longitudinal analysis ###

## create bounds for SSCI imputations (restrict to values of 4-20) and PHQ-9 values at follow-up (restrict to values 0-27)

bds_long <- matrix(c(15,12,4,0,20,27), nrow=2, ncol=3)

bds_long ##check that it worked

## use AMELIA for 30 multiple imputations

set.seed(71086)

m = 30

INDEP_long_imp <- amelia(INDEP_long, m=m, p2s = 1,

idvars=c("PID","PType","SID"),

noms=c("Stigma_cond","time"),

ords=c("educ", "Sex", "hh_inc"),

max.resample = 1000,

bounds = bds_long)

INDEP_long_imp

plot(INDEP_long_imp) ## assess convergence ## education variable has multiple peaks indicating overdispersion

write.amelia(obj=INDEP_long_imp, file.stem="INDEP_long_imp")

## set up imputed datasets for longitudinal analysis, store as list-like object

ll.long.imp_sets <-

lapply(1:m, function(i){

INDEP_long_imp.i <- read.csv(paste0('indep_long_imp', i, '.csv'), header=TRUE) ## load m=30 imputed datasets

INDEP_long_imp.i$PHQ9_tot.y <-round(INDEP_long_imp.i$PHQ9_tot.y) ## round imputed PHQ9 values to nearest whole numbers (no missingness at baseline)

INDEP_long_imp.i$SSCI <- round(INDEP_long_imp.i$SSCI) ## round imputed SSCI values to nearest whole number

INDEP_long_imp.i$A1C.y <- round(INDEP_long_imp.i$A1C.y, digits = 1) ## round imputed A1c values to 1 decimal place (no missingness at baseline)

return(INDEP_long_imp.i)

})

## check an imputed dataset

INDEP_long_imp1 <- ll.long.imp_sets[[1]]

### longitudinal analysis of SSCI by treatment group ###

## model formula

## intervention assignment = predictor, total self-stigma score as outcome

mod_long <- SSCI ~ PType + as.numeric(time) + PType*as.numeric(time) + Age + Sex + as.factor(hh_inc) + as.factor(educ) + PHQ9_tot.x + A1C.x

## mixed effects model, intervention*time interaction, random intercept for individuals with different SSCI values, exchangeable correlation structure, no random slopes

ll.long.mod <-

lapply(1:m, function(i) {

mod.long.outp <- lme(mod_long,

random = ~ 1 | PID,

correlation = corCompSymm(form = ~1 | PID),

method = "ML",

data = ll.long.imp_sets[[i]])

})

ll.long.mod

## pool results together

long.fit <- pool(ll.long.mod)

## point estimates, robust CI, p values

summary(long.fit, conf.int=TRUE)

## random effects model parameters

testEstimates(ll.long.mod, var.comp=TRUE, extra.pars = TRUE)
