# Supplementary material for: A mediation analysis evaluating change in self-stigma on diabetes outcomes among people with depression in urban India: A secondary analysis from the INDEPENDENT trial of the collaborative care model
Source: PLOS Glob Public Health. 2024 Sep 4;4(9):e0003624. doi: 10.1371/journal.pgph.0003624 (PMC11373850; doi:10.1371/journal.pgph.0003624)
Supplement: Attachment — Submitted filename: Mediation_07.18.2024_response to reviewers.docx [file pgph.0003624.s004.docx]

July 18, 2024

Dear PLOS Global Public Health editors,

Thank you so much to the peer reviewer for their helpful feedback and comments on our manuscript “A mediation analysis evaluating change in self-stigma on diabetes outcomes among people with depression in urban India: A secondary analysis from the INDEPENDENT trial of the Collaborative Care Model” (PGPH-D-24-00397). We are also grateful for your editorial responses and support for our manuscript. We detail the changes we’ve made for our resubmission in the response letter below.

Inclusivity in Global Research Checklist:

*We have included an Inclusivity Checklist with our resubmission. Pursuant to the directions in the checklist, we have added content to the manuscript including a line on protocol deviations in the Data sources and participants subsection of the Methods and a line in the Ethics subsection of the Methods to direct readers to the checklist as a supplemental file. Thank you for your strong commitment to innovating and promoting best practices in authorship and research ethics in research globally.*

**Reviewer 1:**

General comments:

*Thank you so much for your thorough review and helpful comments, which will result in an improved manuscript.*

-p3, line 57-58 ‘’Importantly, negative attitudes and beliefs associated with self-stigma can be reinforced by healthcare providers..’’ It is presented as something commonly known, but I believe there are strong cultural differences in how healthcare providers could reinforce self-stigma. Could you further explain what you mean by this, in your Indian context?

*Thank you for the suggestion. Pursuant to your next comment below, we’ve elaborated more on the process of how healthcare providers stigmatize mental disorders in the Indian context in the next paragraph. Here, in the global context, we’ve provided general pathways through which providers contribute to self-stigma with some examples.*

-p3, line 62-63 ‘’Visiting a specialist mental health care provider….mental disorders’.’ Could you further explain the mechanism behind or link between visiting mental healthcare providers and stereotyping, prejudice and discrimination?

*Thank you and we agree that elaborating on this would be helpful. We’ve provided an additional sentence explanation here in text, which we hope will help clarify the components about how healthcare providers in India can stigmatize mental disorders (e.g., limited training, medication availability, lack of privacy and importantly, reinforcement of misconceptions/attitudes). In doing so and after re-checking references, we also make the important distinction that 1) these contribute to the stigmatization of mental disorders more generally and not only to stereotyping, prejudice, and discrimination and 2) that primary care providers are often the source of this stigmatization process and not just specialty providers. We also added an additional line in the discussion – explaining one important study, where patients with schizophrenia experienced internalized stigma more than communal stigma – to further contextualize this point.*

-In the introduction it is not clear what type of diabetes participants were diagnosed with. Is it all type 2 diabetes? Or type 1 diabetes as well?

*Thank you and this is an important point. We have clarified in the Introduction and Methods sections that these were exclusively participants with type 2 diabetes.*

Methods:
-p4, line 95-100 ‘’In this pragmatic trial ….. 36 months’’. This is a very long and difficult to read sentence. Could you change it into several shorter sentences?

*Thank you and we have done so accordingly.*

-p4, line 100 ‘’To be eligible, participants must have been 35 years…’’ Why 35? Could you explain why younger adults would not be included? -> see also my comment on type of diabetes (introduction), that might be related to this.

*Thank you. There was a consensus discussion among the primary trial investigators resulting in this eligibility criteria to improve enrollment flow because type 2 diabetes (pursuant to other comments) is uncommon under age 35 and as the recruitment required screening for depressive symptoms.*

-p.7, Figure 1: Why did the authors chose to compare changes in self-stigma to HbA1c at 12 months? Why not look at the relationship between change in self-stigma and change in HbA1c and change in depression? I am no statistician, but conceptually I would think that it makes sense to compare changes in all 3 variables. Especially since there seems to be quite a change in depression scores and HbA1c from baseline to 12 months.

*Thank you for this thoughtful comment. This secondary analysis was done using data from a primary trial comparing the effectiveness of an integrated care intervention at improving diabetes and depression outcomes over 12 months, which accounts for the changes you noticed. We proposed that one critical part of why the intervention was effective was that the intervention may have improved self-stigma, which we wanted to evaluate in the longitudinal analysis. And that such improvements in turn were responsible for predicting the mediated pathway. However, your comment illustrates we could have articulated this more clearly. We've added the following in the Methods/Data Analysis subsection:* *[line 159] "We propose that an important part of why CoCM was effective at improving diabetes and depression outcomes was because CoCM led to improvements in self-stigma, and this change in turn predicted the patient clinical outcomes of interest". We also added the following at line 158 to clarify the ANCOVA approach for adjusting for baseline values of the mediator and outcome variables (as opposed to the predictor where we were explicitly interested in the change score as a predictor) in lieu of change scores given the data came from a trial: "Given the primary trial design and to account for depressive symptom severity... accordingly instead of using change scores for both which conserved power and improved statistical precision."* *The van Breukelen article cited in-text elaborates further on the rationale for conserved power and improved precision through the ANCOVA approach.*

Results:
-p8, line 173: ‘’and indicated diabetes as their most stigmatizing condition’’ = double, please remove.

*Thank you for this catch and we’ve modified it accordingly.*

Once again, our appreciation for all the support for our manuscript and we look forward to the review of our resubmission. Please do not hesitate to contact me if you need more information.

Sincerely,

Scott Halliday, PhD
